# Association between lower plasma adiponectin levels and higher liver stiffness in type 2 diabetic individuals with nonalcoholic fatty liver disease: an observational cross-sectional study

**DOI:** 10.1007/s42000-022-00387-6

**Published:** 2022-07-13

**Authors:** Alessandro Mantovani, Chiara Zusi, Alessandro Csermely, Gian Luca Salvagno, Antonio Colecchia, Giuseppe Lippi, Claudio Maffeis, Giovanni Targher

**Affiliations:** 1 Section of Endocrinology, Diabetes and Metabolism, Department of Medicine, University of Verona, Piazzale A. Stefani, 1, 37126 Verona, Italy; 2 Pediatric Diabetes and Metabolic Disorders Unit, Department of Surgical Sciences, Dentistry, Pediatrics, and Gynaecology, University of Verona, Verona, Italy; 3 Section of Clinical Biochemistry, Department of Medicine, University of Verona, Verona, Italy; 4 Gastroenterology Unit, Department of Medical Specialties, University of Modena & Reggio Emilia and Azienda Ospedaliero, Universitaria Di Modena, Modena, Italy

**Keywords:** Hypoadiponectinemia, Liver fibrosis, NAFLD, Nonalcoholic steatohepatitis, NASH, Type 2 diabetes

## Abstract

**Purpose:**

Little is known about the association between plasma adiponectin levels and nonalcoholic fatty liver disease (NAFLD) in patients with type 2 diabetes mellitus (T2DM). We examined whether there is an association between lower plasma adiponectin levels and the presence/severity of NAFLD in people with T2DM.

**Methods:**

We cross-sectionally recruited 79 men with non-insulin-treated T2DM and no known liver diseases, who had consecutively attended our diabetes outpatient service over a 6-month period and who underwent both ultrasonography and Fibroscan-measured liver stiffness (LSM). Nine single nucleotide polymorphisms (*PNPLA3* rs738409 and other genetic variants) associated with NAFLD were investigated.

**Results:**

Among the 79 participants included (mean age 67 ± 10 years, BMI 27.7 ± 4 kg/m^2^), 28 did not have NAFLD, 32 had steatosis alone, and 19 had NAFLD with coexisting significant fibrosis (LSM ≥ 7.0 kPa by Fibroscan®). Compared to those without NAFLD, patients with hepatic steatosis alone and those with hepatic steatosis and coexisting significant fibrosis had lower high-molecular-weight adiponectin levels (5.5 [IQR 2.3–7.6] *vs*. 2.4 [1.8–3.7] vs. 1.6 [1.0–2.9] µg/mL; *p* < 0.001). After adjustment for age, body mass index, insulin resistance, and the *PNPLA3* rs738409 variant, lower plasma adiponectin levels were found to be associated with increased odds of both steatosis alone (adjusted-odds ratio [OR] 2.44, 95% CI 1.04–5.56, *p* = 0.042) and NAFLD with coexisting significant fibrosis (adjusted-OR 3.84, 95% CI 1.23–10.0, *p* = 0.020). Similar findings were observed after adjustment for the other eight genotyped NAFLD-related polymorphisms.

**Conclusion:**

Lower plasma adiponectin levels are closely associated with the presence and severity of NAFLD in men with T2DM, pointing to a role of adiponectin in NAFLD development and progression.

**Supplementary Information:**

The online version contains supplementary material available at 10.1007/s42000-022-00387-6.

## Introduction

In the last few years, there has been steadily increasing scientific interest in nonalcoholic fatty liver disease (NAFLD) and nonalcoholic steatohepatitis (NASH) because of their growing burden on public health worldwide [1, 2]. NAFLD has reached epidemic proportions in most high-income countries, affecting up to a third of the general adult population [3, 4] and up to ~ 70% of patients with type 2 diabetes mellitus (T2DM) [5], as well as almost all patients with severe obesity [6].

Although the precise mechanisms involved in the development and progression of NAFLD remain to be fully elucidated, it has become increasingly clear that insulin resistance and low-grade inflammation are two key factors involved in the pathophysiology of NAFLD [7]. Crosstalk between adipokines and proinflammatory cytokines may play a role in the development of this liver disease [8, 9]. Adiponectin is a hormone mainly produced by adipocytes, which circulates in three different isoforms (i.e., low-molecular-weight (LMW), middle-molecular-weight (MMW), and high-molecular-weight (HMW) adiponectin) in the bloodstream [8–10]. Specifically, circulating levels of adiponectin are closely and inversely associated with insulin resistance, excess body weight, and ectopic fat deposition [8, 9].

In recent years, many observational studies [11–16] and some meta-analyses [17] have reported that lower plasma adiponectin levels are significantly associated with the presence and severity of NAFLD, especially in patients without T2DM, thereby suggesting that hypoadiponectinemia might represent a risk factor for NAFLD. However, little information is available to date on the association between lower plasma adiponectin levels and the presence of NAFLD or NASH in patients with T2DM [18]. Nor, to our knowledge, are any data available assessing whether this association is at least partially mediated by coexistence of the rs738409 genetic variant in the patatin-like phospholipase domain-containing 3 (*PNPLA3*) gene or other genetic polymorphisms that confer greater susceptibility to NAFLD and NASH [19, 20].

Therefore, in this cross-sectional study, we examined the following: (i) whether there was an association between plasma adiponectin levels and the presence and severity of NAFLD (as detected by liver ultrasonography and vibration-controlled transient elastography [VCTE]) in ambulatory patients with established T2DM and (ii) whether this association could be, at least partly, mediated by the coexistence of the *PNPLA3* rs738409 variant or other NAFLD-related genetic polymorphisms.

## Methods

### Patients

We studied 79 Caucasian male volunteers with non-insulin-treated T2DM who had consecutively attended our diabetes outpatient service over a period of 6 months (from January to June 2019) and who agreed to undergo both liver ultrasonography and VCTE for diagnosis and staging of NAFLD. The exclusion criteria of the study were as follows: (a) history of significant alcohol consumption (defined as > 20 g of alcohol per day) and other known causes of chronic liver diseases (e.g., virus, drugs, autoimmunity, and hemochromatosis); (b) cirrhosis, cancer, and end-stage kidney disease (defined as estimated GFR < 15 mL/min/1.73 m^2^ or chronic dialysis); (c) chronic use of potentially hepatotoxic drugs; and (d) insulin treatment. Taking into account the technical limitations of VCTE, patients with free abdominal fluid or congestive heart failure were also excluded from the study. Most patients enrolled in this study have also been included in other published studies [21, 22].

### Clinical and laboratory data

Body mass index (BMI) was measured as kilograms divided by the square of height in meters. Waist circumference was measured at the midpoint between the lowest rib and the iliac crest. Blood pressure was measured with a standard sphygmomanometer after the subject had been seated quietly for at least 5 min. Subjects were considered to have arterial hypertension if their blood pressure was ≥ 140/90 mmHg or if they were taking any antihypertensive agents.

Venous blood samples were collected in the morning after an overnight fast. Complete blood count, serum liver enzymes (aspartate aminotransferase [AST], alanine aminotransferase [ALT], gamma-glutamyltransferase [GGT]), glucose, lipids, and other biochemical blood parameters were measured using standard laboratory procedures at the Central Laboratory of Verona Hospital, Verona, Italy. Hemoglobin A1c (HbA1c) was measured using the high-performance liquid chromatography analyzer Tosoh-G7 (Tosoh Bioscience Inc., Tokyo, Japan). Fasting insulin concentrations were measured using a chemiluminescent immunoassay (LIAISON, DiaSorin, Saluggia, Italy). Homeostasis model assessment (HOMA-IR) score was used for estimating insulin resistance [23]. The measurement of plasma adiponectin concentration was performed in duplicate by using an automated two-step sandwich immunoassay method specific for high-molecular-weight adiponectin via chemiluminescent enzyme immunoassay technology (Lumipulse® G HMW-Adiponectin, Fujirebio, Tokyo, Japan). The intra-assay and inter-assay coefficients of variation were < 2.5 and < 3.3%, respectively [21]. Glomerular filtration rate (e-GFR) was estimated using the Chronic Kidney Disease Epidemiology Collaboration (CKD-EPI) equation [24].

The presence of ischemic heart disease was defined as a documented history of myocardial infarction, angina, or coronary revascularization procedures. The presence of diabetic retinopathy (diagnosed with fundoscopy after pupillary dilation) was also recorded.

### Liver ultrasonography and VCTE

Liver ultrasonography and VCTE were performed by a single expert physician, who was blinded to participants’ clinical and biochemical details. Ultrasound liver images were obtained with the participant (in the fasting state) on the left side in a decubitus position, with their right arm stretched above the head after taking a deep breath. Hepatic steatosis was diagnosed by ultrasonography using a 4-MHz probe (MyLab 70, Esaote Group, Genova, Italy) according to specific ultrasound characteristics, including diffuse hyperechogenicity of the liver relative to the kidneys, ultrasound beam attenuation, and poor visualization of intra-hepatic vessel borders and the diaphragm [22, 25, 26]. Radiological scoring systems (e.g., the Hamaguchi index) and semi-quantitative ultrasonographic indices of steatosis severity were not available in this study.

Liver stiffness measurement (LSM) was performed by VCTE using Fibroscan® (Echosens, Paris, France) and an M probe. We did not have the Fibroscan® XL probe for severely obese patients. The accuracy of the Fibroscan® M probe to identify significant liver fibrosis is excellent in patients with overweight or grade 1 obesity (BMI ≤ 35 kg/m^2^) [27]. In our study, only three patients had a BMI > 35 kg/m^2^. Our Fibroscan® system was not equipped with the controlled attenuation parameter (CAP) technology for measuring hepatic steatosis. A trained physician performed LSM examinations in all patients after at least 8 h of fasting and after liver ultrasonography. Additional details of the examination procedures have been described elsewhere [25]. Briefly, each patient’s LSM was considered adequate if it included at least 10 valid measurements, with a success rate > 60% and measurement variability < 30% of the median [22]. The presence of significant hepatic fibrosis was defined as the presence of LSM ≥ 7 kPa (corresponding to Kleiner fibrosis stage ≥ F2 on liver histology) [22].

### Genetic analysis

Genomic DNA was extracted from peripheral blood leukocytes using the QIAamp DNA Blood Mini Kit (QIAGEN GmbH, Hilden, Germany). In all participants, genotyping was carried out by predesigned TaqMan probes (Applied Biosystems, Foster City, CA, USA), according to the manufacturer’s protocol. Polymorphism genotyping was performed using 7900 HT Real-Time PCR (Applied Biosystems, USA). We genotyped the following nine genetic polymorphisms that have been associated with greater susceptibility to NAFLD or NASH: rs738409 in the patatin-like phospholipase domain-containing protein-3 (*PNPLA3*) gene, rs58542926 in the trans-membrane 6 superfamily member 2 (*TM6SF2*) gene, rs641738 in the membrane-bound O-acyltransferase domain containing 7 (*MBOAT7*) gene, rs1260326 in the glucokinase regulatory protein (*GCKR*) gene, rs2236212 in the elongation of very-long-chain fatty acids-like 2 (*EVLOV2*) gene, rs1535 in the fatty acid desaturase 2 (*FADS2*) gene, rs13412852 in the lipin 1 (*LPIN1*) gene, rs1800591 in the microsomal triglyceride transfer protein (*MTTP*) gene, and rs4880 in the superoxide dismutase 2 (*SOD2*) gene, respectively.

### Statistical analysis

Owing to the exploratory design of the study, we did not perform an a priori sample size calculation. Continuous variables were expressed as means ± SD or medians and interquartile range (IQR) when indicated, while categorical variables were expressed as proportions. The chi-square test for categorical variables, the one-way analysis of variance for normally distributed continuous variables, and the Kruskal–Wallis test for non-normally distributed continuous variables (i.e., diabetes duration, triglycerides, insulin, HOMA-IR score, adiponectin, and Fibroscan-assessed LSM) were used to examine the intergroup differences in the main clinical and biochemical characteristics of participants, who were simultaneously stratified by the presence and severity of NAFLD (Table 1).

**Table 1:** Clinical, biochemical, and genetic characteristics of men with type 2 diabetes, stratified by presence and severity of NAFLD (using both liver ultrasonography and Fibroscan®)

	Patients without hepatic steatosis (*n* = 28)	Patients with hepatic steatosis alone (*n* = 32)	Patients with hepatic steatosis and significant fibrosis (*n* = 19)	*p* value
Age (years)	71.3 ± 8	64.8 ± 8	62.8 ± 10	**0.002**
Weight (kg)	76.8 ± 14	86.9 ± 15	91.5 ± 10	**0.001**
BMI (kg/m^2^)	25.8 ± 4	28.2 ± 4	29.8 ± 4	**0.002**
Waist circumference (cm)	97 ± 10	103 ± 13	108 ± 10	**0.004**
Diabetes duration (years)	13.5 (6–22)	10 (7–18)	9 (4–15)	0.275
Current smokers (%)	10.7	12.5	15.8	0.913
Systolic blood pressure (mmHg)	131 ± 20	133 ± 20	131 ± 16	0.863
Diastolic blood pressure (mmHg)	72 ± 12	77 ± 8	82 ± 9	**0.005**
Platelet count (× 100,000/mm^3^)	221 ± 66	222 ± 52	234 ± 69	0.743
Fasting glucose (mmol/L)	7.2 ± 1.8	7.1 ± 1.3	7.4 ± 1.6	0.779
Hemoglobin A1c (mmol/mol Hb)	51.3 ± 8.7	53.7 ± 6.2	53.1 ± 8.4	0.475
Fasting insulin (mU/L)	4.1 (2.7–7.2)	6.0 (3.8–9.1)	9.6 (4.2–16.8)	**0.001**
HOMA–IR score	1.3 (0.7–1.9)	1.8 (1.1–2.8)	3.2 (1.3–5.9)	** < 0.001**
Total cholesterol (mg/dL)	163 ± 45	141 ± 35	149 ± 30	0.083
HDL cholesterol (mg/dL)	51 ± 11	48 ± 16	43 ± 11	0.167
Triglycerides (mg/dL)	92 (69–144)	106 (89–138)	158 (124–209)	**0.002**
AST (IU/L)	24 ± 5	27 ± 11	27 ± 8	0.289
ALT (IU/L)	13 ± 5	17 ± 8	18 ± 9	**0.022**
GGT (IU/L)	19 (14–29)	22 (15–34)	30 (17–50)	0.064
Albumin (g/dL)	48 ± 3	49 ± 2	48 ± 3	0.197
Creatinine (umol/L)	91.7 ± 27	81.2 ± 22	89.4 ± 17	0.199
e-GFR_CKD-EPI_ (mL/min/1.73 m^2^)	83.1 ± 28	96.2 ± 29	83.3 ± 19	0.107
Hypertension (%)	85.7	75.0	79.0	0.596
Ischemic heart disease (%)	14.3	12.5	21.1	0.671
Diabetic retinopathy, any degree (%)	15.4	20.0	11.1	0.788
Metformin (%)	75.0	96.9	100	**0.005**
Sulfonylureas (%)	17.9	28.1	36.9	0.320
Pioglitazone (%)	17.9	15.6	10.5	0.855
DPP-4 inhibitors (%)	14.3	28.1	21.1	0.448
GLP-1 receptor analogues (%)	21.4	28.1	31.6	0.767
SGLT-2 inhibitors (%)	3.6	21.9	21.1	0.093
Antiplatelet drugs (%)	44.4	58.1	42.1	0.500
Beta-blockers (%)	40.7	22.6	36.8	0.322
ARB or ACE inhibitors (%)	66.7	58.1	57.9	0.798
Calcium-channel blockers (%)	25.9	25.8	36.9	0.659
Diuretics (%)	25.9	22.6	31.6	0.758
Statins (%)	85.7	83.9	68.4	0.312
HMW adiponectin (ug/mL)	5.5 (2.3–7.6)	2.4 (1.8–3.7)	1.6 (1.0–2.9)	** < 0.001**
Fibroscan-assessed LSM (kPa)	4.5 (3.7–5.3)	5.5 (4.7–6.2)	8.8 (7.9–11.8)	ND
*PNPLA3* rs738409				0.973
CC (%)	46.4	50.0	52.6	
CG (%)	50.0	43.8	42.1	
GG (%)	3.6	6.3	5.3	
*TM6SF2* rs58542926				0.525
CC (%)	89.3	84.4	94.7	
CT (%)	10.7	15.6	5.3	
TT (%)	0	0	0	
*MBOAT7* rs641738				0.754
CC (%)	32.1	60.7	7.1	
CT (%)	31.3	53.1	15.6	
TT (%)	31.7	63.2	5.3	

Bold entries in *P*-value are statistically significant

Sample size, *n* = 79. Data are expressed as means ± SD, medians, and IQR (in parentheses) or relative percentages. Differences among the three patient groups were tested by the one-way ANOVA for normally distributed variables, the Kruskal–Wallis test for non-normally distributed variables (i.e., diabetes duration, triglycerides, GGT, fasting insulin, HOMA-IR score, adiponectin), or the chi-square test for categorical variables. Hypertension was defined as blood pressure ≥ 140/90 mmHg and/or drug treatment

*Abbreviations*: *ACE* angiotensin-converting enzyme, *ALT* alanine aminotransferase, *ARB* angiotensin II receptor blocker, *AST* aspartate aminotransferase, *BMI* body mass index, *DPP-4* dipeptidyl peptidase-4, *e-GFR* estimated glomerular filtration rate, *GGT* gamma-glutamyltransferase, *GLP-1* glucagon-like peptide-1, *HMW* high molecular weight, *HOMA-IR* homeostasis model assessment-insulin resistance, *MBOAT7* membrane-bound O-acyltransferase domain-containing 7, *PNPLA3* patatin-like phospholipase domain-containing protein 3, *SGLT-2* sodium/glucose cotransporter-2, *TM6SF2* transmembrane 6-superfamily member 2, *ND* not determined

We tested the independent association between plasma adiponectin concentrations and the presence/severity of NAFLD by using unadjusted and adjusted multinomial logistic analyses. The dependent variable for these multinomial logistic regression models was the presence and severity of NAFLD, categorized as follows: patients without hepatic steatosis on ultrasound (reference group), patients with hepatic steatosis alone (group 1), and patients with hepatic steatosis and coexisting significant liver fibrosis, defined as LSM ≥ 7 kPa on VCTE (group 2). As depicted in Table 2, we performed four adjusted multinomial logistic regression models. Model 1 was adjusted for age, BMI (or waist circumference), and HOMA-IR score; model 2 was adjusted for age, BMI (or waist circumference), HOMA-IR score, and the *PNPLA3* rs738409 variant; model 3 was controlled for age, BMI (or waist circumference), HOMA-IR score, and the *TM6SF2* rs58542926 variant; and, finally, model 4 was controlled for age, BMI (or waist circumference), HOMA-IR score, and the *MBOAT7* rs641738 variant. We also performed six other adjusted multinomial logistic models that adjusted for the same covariates of model 2 plus the other six genotyped NAFLD-related genetic variants (see supplementary material). The impact of each genetic variant on the severity of NAFLD was assessed using dominant genetic models. All the aforementioned multinomial logistic regression models were repeated even after excluding patients treated with pioglitazone, which is a glucose-lowering drug able to increase the circulating levels of adiponectin and improve liver fibrosis [15, 28], or those treated with SGLT2-inhibitors or GLP-1 receptor agonists, which are two glucose-lowering drugs also able to improve hepatic steatosis and increase plasma adiponectin levels. Covariates included in these logistic regression models were selected as potential confounding factors based on their significance in univariable analyses or based on their biological plausibility.

**Table 2:** Association between lower levels of high-molecular-weight adiponectin and presence and severity of NAFLD in patients with type 2 diabetes

	Patients without hepatic steatosis (*n* = 28)	Patients with hepatic steatosis alone (*n* = 32)			Patients with hepatic steatosis and significant fibrosis (*n* = 19)		
		Odds ratio(s)	95% CI	*p* value	Odds ratio(s)	95% CI	*p* value
Unadjusted model							
Decrease in adiponectin (ug/mL)	*Ref*	3.12	1.45–6.67	**0.004**	6.25	2.43–16.7	** < 0.001**
Adjusted model 1							
Decrease in adiponectin (ug/mL)	*Ref*	2.43	1.03–5.56	**0.043**	3.70	1.23–11.1	**0.020**
Age (years)	*Ref*	0.95	0.88–1.02	0.143	0.94	0.86–1.03	0.194
Body mass index (kg/m^2^)	*Ref*	1.13	0.94–1.36	0.183	1.14	0.91–1.43	0.262
HOMA-IR score	*Ref*	1.02	0.60–1.76	0.934	1.47	0.84–2.59	0.179
Adjusted model 2							
Decrease in adiponectin (ug/mL)	*Ref*	2.44	1.04–5.56	**0.042**	3.84	1.23–10.0	**0.020**
Age (years)	*Ref*	0.95	0.88–1.02	0.144	0.94	0.86–1.03	0.193
Body mass index (kg/m^2^)	*Ref*	1.13	0.94–1.37	0.181	1.14	0.90–1.44	0.263
HOMA-IR score	*Ref*	1.03	0.60–1.78	0.916	1.48	0.83–2.63	0.181
* PNPLA3* rs738409 (CC vs. CG/GG genotype)	*Ref*	1.20	0.43–3.29	0.729	1.22	0.34–4.32	0.759
Adjusted model 3							
Decrease in adiponectin (ug/mL)	*Ref*	2.56	1.08–6.25	**0.034**	4.00	1.29–14.3	**0.015**
Age (years)	*Ref*	0.94	0.87–1.01	0.115	0.94	0.86–1.03	0.184
Body mass index (kg/m^2^)	*Ref*	1.17	0.96–1.42	0.120	1.16	0.91–1.47	0.225
HOMA-IR score	*Ref*	0.94	0.55–1.62	0.824	1.37	0.78–2.39	0.272
* TM6SF2* rs58542926 (CC vs. CT genotype)	*Ref*	3.59	0.49–26.7	0.210	1.30	0.08–21.1	0.855
Adjusted model 4							
Decrease in adiponectin (ug/mL)	*Ref*	2.38	1.02–5.56	**0.045**	3.58	1.29–11.1	**0.024**
Age (years)	*Ref*	0.94	0.88–1.02	0.132	0.94	0.86–1.03	0.177
Body mass index (kg/m^2^)	*Ref*	1.14	0.95–1.38	0.162	1.13	0.90–1.43	0.282
HOMA-IR score	*Ref*	1.01	0.58–1.74	0.984	1.48	0.84–2.63	0.179
* MBOAT7* rs641738 (CC vs. CT/TT genotype)	*Ref*	1.43	0.57–3.62	0.447	0.97	0.29–3.29	0.962

Bold entries in *P*-value are statistically significant

Sample size, *n* = 79 unless where indicated. Data are expressed as odds ratio and 95% confidence intervals (CI) as tested by multinomial logistic regression analysis. The dependent variable for all multinomial logistic regression models was the presence and severity of NAFLD, categorized as follows: patients without hepatic steatosis on ultrasound (reference group), patients with hepatic steatosis alone (group 1), and patients with steatosis and coexisting significant fibrosis on VCTE (group 2). Plasma adiponectin and HOMA-IR values were logarithmically transformed before analysis. The impact of each genetic variant on the presence and severity of NAFLD was assessed using dominant genetic models

*Abbreviations*: *HOMA-IR* homeostasis model assessment-insulin resistance, *MBOAT7* membrane-bound O-acyltransferase domain containing 7, *PNPLA3* patatin-like phospholipase domain-containing protein 3, *ref.* reference category, *TM6SF2* transmembrane 6 superfamily member 2

All statistical tests were two-sided, and a *p* value of < 0.05 (two tailed) was considered statistically significant. Statistical analyses were performed using STATA software, version 16.1 (STATA, College Station, TX, USA).

## Results

Of the 79 men with non-insulin-treated T2DM included in the study (mean age 67 ± 10 years, BMI 27.7 ± 4 kg/m^2^, HbA1c 52 ± 6 mmol/mol), 28 (35.5%) did not have NAFLD on ultrasonography, 32 (40.5%) had hepatic steatosis alone, and 19 (24%) had NAFLD with coexisting significant fibrosis (i.e., LSM ≥ 7.0 kPa on Fibroscan®), respectively.

Table 1 shows the main clinical, biochemical, and genetic characteristics of participants who were simultaneously stratified by the presence and severity of NAFLD. Compared with those without NAFLD, patients with NAFLD, regardless of the presence or absence of significant fibrosis, were more likely to be younger, centrally obese, and more insulin resistant (as reflected by a higher HOMA-IR score) and also had greater diastolic blood pressure and higher serum ALT and triglyceride concentrations. Notably, compared to those without NAFLD, patients with NAFLD had markedly lower high-molecular-weight adiponectin levels (5.5 [IQR 2.3–7.6] vs. 2.4 [1.8–3.7] vs. 1.6 [1.0–2.9] μg/mL; *p* < 0.001 for trend). No significant intergroup differences were observed in terms of diabetes duration, smoking history, systolic blood pressure, glycemic control, total and HDL cholesterol, platelet count, AST, GGT, albumin, kidney function parameters, and comorbidities (such as prior ischemic heart disease and diabetic retinopathy), as well as the current use of lipid-lowering, anti-hypertensive, antiplatelet, or glucose-lowering agents (except for metformin use). Moreover, there were no significant intergroup differences in the distribution of the *PNPLA3*, *TM6SF2*, and *MBOAT7* genetic variants among the patient subgroups. No significant intergroup differences were also found in the other six genotyped NAFLD-related polymorphisms (data not shown).

**Fig. 1:**
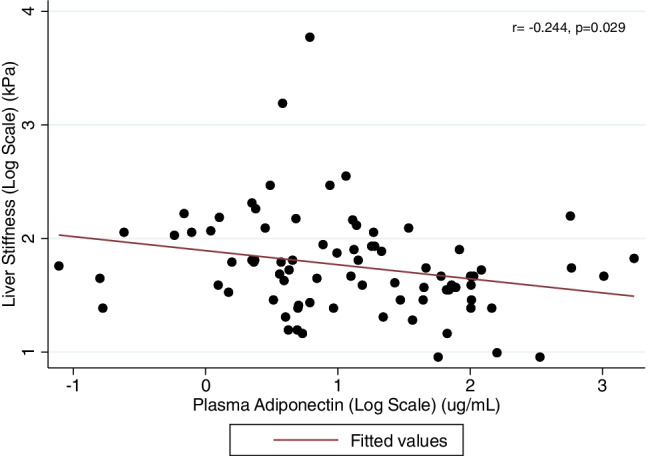
Univariable linear correlation between liver stiffness measurement (LSM) (by using Fibroscan®) and plasma high-molecular-weight adiponectin levels in men with T2DM. Both LSM and plasma adiponectin values were logarithmically transformed before analysis

Figure 1 shows that there was a significant inverse association between plasma adiponectin levels and Fibroscan-assessed LSM in the whole group of patients.

The independent association between plasma adiponectin levels and the presence and severity of NAFLD is illustrated in Table 2. In the unadjusted regression model, lower plasma adiponectin levels were significantly associated with nearly threefold and sixfold odds of either hepatic steatosis alone or NAFLD with coexisting significant fibrosis. These results remained statistically significant after adjustment for age, BMI, and HOMA-IR score (model 1). The additional adjustment for either the *PNPLA3* (model 2), *TM6SF2* (model 3), or *MBOAT7* (model 4) genetic variants did not substantially change the results. Almost identical results were also observed even when we included waist circumference as a covariate (instead of BMI), when we additionally adjusted for duration of diabetes, or when we excluded those with Fibroscan-assessed LSM ≥ 20 kPa (*n* = 2) (data not shown). Similar results were also observed when we excluded patients treated with pioglitazone (*n* = 13) (Supplementary Table 1), those treated with SGLT-2 inhibitors (*n* = 12) (Supplementary Table 2), or those treated with GLP-1 receptor agonists (*n* = 21) (Supplementary Table 3). However, in this latter case, the significance of the results was weaker in fully adjusted regression models; we believe that this is likely due to the smaller sample size available for this latter analysis (58 patients included). Finally, as reported in Supplementary Table 4, the further adjustment for the other six genotyped NAFLD-related polymorphisms did not change the results of the study.

## Discussion

The main findings of our cross-sectional study are as follows: (a) in Caucasian men with non-insulin-treated T2DM, lower plasma high-molecular-weight adiponectin levels were significantly associated with ~ threefold and sixfold odds of either hepatic steatosis alone or NAFLD with coexisting significant fibrosis, and (b) these associations remained statistically significant even after adjusting for age, adiposity measures, duration of diabetes, HOMA-IR score, the *PNPLA3* rs738409 variant, or other genetic variants associated with greater susceptibility of NAFLD and fibrosing NASH.

In a meta-analysis of 27 observational studies that reported data on 2243 individuals (1545 patients with NAFLD and 698 controls), Polyzos et al. reported that control subjects had significantly higher plasma adiponectin levels compared to patients with simple steatosis alone (random-effects weighted mean difference [WMD] 3.0, 95% CI 1.57–4.43, *I*^2^ = 80.4%) or those with NASH (random-effects WMD 4.70, 95% CI 3.71–5.78, *I*^2^ = 84.1%) [17]. By performing a meta-regression analysis, age, sex, BMI, and pre-existing diabetes failed to account for the observed heterogeneity [17]. In another observational study, plasma adiponectin levels were significantly lower in patients with NAFLD than in those with viral hepatitis or other chronic liver diseases [29]. In a cross-sectional study including 84 Brazilian obese patients with T2DM and biopsy-proven NAFLD, Leite et al. reported that plasma adiponectin levels were lower in patients with NASH than in their counterparts without NASH [18]. Finally, in a recent systematic review and meta-analysis of 12 case–control studies, Zheng et al. reported that rs266729 and rs3774261 polymorphisms in the adiponectin gene were associated with a higher risk of having NAFLD among Asian, Chinese, and Caucasian populations [30].

Collectively, our findings confirm and extend the aforementioned observations, showing a strong association between lower plasma adiponectin levels and the presence and severity of NAFLD (as assessed with liver ultrasonography and Fibroscan®, which are the two most widely used diagnostic tools to non-invasively diagnose and stage NAFLD in clinical practice [31]) in Caucasian outpatients with non-insulin-treated T2DM. Some studies showed that NAFLD patients carrying the *PNPLA3* G/G genotype had lower plasma adiponectin levels compared to those with the *PNPLA3* C/C genotype [32], whereas others did not [14]. Notably, in our study, we showed for the first time that the inverse association between plasma adiponectin levels and the presence/severity of NAFLD remained statistically significant even after adjusting for the *PNPLA3* rs738409 variant or other less common NAFLD-related genetic polymorphisms. Although the results of our study do not have clear implications for clinical practice, they may be important for guiding future mechanistic and intervention studies. In fact, the evidence from this and other studies [11–16, 18] suggests a possible role of decreased adiponectin levels in the pathophysiology of NAFLD, thus underlining the need for future large-scale studies assessing the predictive as well as the therapeutic role of this adipokine in the spectrum of NAFLD. Additionally, these results may encourage the designing of diagnostic accuracy studies to examine whether plasma adiponectin levels may contribute (alone or in combination with other biomarkers) to the achievement of a non-invasive diagnosis of liver fibrosis.

To date, the role of adiponectin in the pathogenesis of NAFLD has not been fully elucidated. It is well known that adiponectin is one of the most important and abundant adipose-tissue-secreted adipokines [33]. Adipocytes secrete adiponectin into the bloodstream as three oligomeric complexes, including a low-molecular-weight (LMW) trimer, a medium-molecular-weight (MMW) hexamer, and a high-molecular-weight (HMW) multimer [10]. HMW adiponectin is the predominant isoform in circulation and has been identified as the most active biological isoform. HMW adiponectin exerts beneficial systemic metabolic and anti-inflammatory effects, as it promotes improvements in hepatic and systemic insulin sensitivity, glucose uptake, and lipid metabolism [10]. Adiponectin acts by binding and activating two different receptor isoforms, namely AdipoR1 and AdipoR2, expressed in skeletal muscle and the liver, among other tissues [34, 35]. Decreased plasma adiponectin levels are strongly associated with abdominal obesity, ectopic fat deposition, and greater insulin resistance [8, 9]. However, adiponectin may also signal in the liver, exerting a beneficial insulin-sensitizing effect [8, 9, 15]. Indeed, adiponectin regulates glucose and lipid metabolism by stimulating hepatic fatty acid oxidation and inhibiting hepatic fatty acid synthesis, mostly via the activation of the AMP-activated protein kinase [15]. Adiponectin may also attenuate liver fibrosis by inducing nitric oxide production of hepatic stellate cells that constitutively express both AdipoR1 and AdipoR2 [36]. Adiponectin also stimulates TIMP metallopeptidase inhibitor-1 (TIMP-1) secretion by hepatic stellate cells to retard their migration, thereby further contributing to the anti-fibrotic effect of adiponectin [37]. Overall, therefore, hypoadiponectinemia might promote the development of NAFLD and liver fibrosis [35, 38]. It is well known that glitazones (especially pioglitazone) have demonstrated promising results in randomized controlled trials for treatment of NASH [39]. Interestingly, parallel increases in plasma adiponectin levels and histological improvement of NASH were also observed in a systematic review of four randomized clinical trials, providing data on 187 patients with NASH treated up to 12 months [40]. Consequently, some authors have proposed that administration of adiponectin or an adiponectin analog (e.g., osmontin) might be an attractive pharmacological strategy for management of conditions characterized by adiponectin deficiency, such as NAFLD or NASH [8, 41]. Another potential therapeutic option would be the upregulation of endogenous adiponectin. Although the use of glitazones, such as rosiglitazone or pioglitazone, has been restricted due to moderate weight gain, peripheral fluid retention, and an increase in myocardial infarction risk (rosiglitazone), the non-thiazolidinedione, selective peroxisome proliferator-activated receptor-γ modulators, like INT131 besylate, could be promising candidates for randomized controlled trials with the potential to improve both glucose metabolism and NAFLD/NASH (while minimizing the side effects of full PPAR-γ agonists) [8, 42]. However, further research is needed to examine the therapeutic role of administration of adiponectin or an adiponectin analog in patients with NAFLD.

Our study has some limitations that should be mentioned. Firstly, the cross-sectional design of this single-center study limits our ability to establish temporal or causal associations between lower adiponectin levels and presence/severity of NAFLD. Secondly, the sample size of our study was small and was comprised of Caucasian men with metabolically well-controlled T2DM. Thus, these results cannot necessarily be extrapolated to other ethnic groups of patients, to women with T2DM (notably, the exploration of sex differences is today a priority of NAFLD research [43]), or to patients with uncontrolled glycemia. On the other hand, this latter limitation can represent a specific strength of our study in that it shows that lower adiponectin levels are associated with NAFLD and significant fibrosis in the absence of major changes in glycemia. Thirdly, we did not perform a liver biopsy or magnetic resonance elastography for staging liver fibrosis. Consequently, we were not able to compare the results of liver stiffness obtained by VCTE with histology data. However, both liver ultrasonography and VCTE are widely used for the diagnosis and staging of NAFLD in routine clinical practice [31]. In addition, a meta-analysis of 12 observational studies (published from January 2011 to February 2021) showed that conventional liver ultrasonography allows for reliable and accurate detection of ≥ 5% histologically defined hepatic steatosis (82% sensitivity and 80% specificity), as well as moderate-severe hepatic steatosis (85% sensitivity and 85% specificity), compared to liver histology [44].

Despite these limitations, our study has some important strengths, including the consecutive enrolment of the study population, its data completeness, and the adjustment for diabetes-related variables and other potential confounders, including a large panel of specific NAFLD-related genetic polymorphisms. In addition, the liver ultrasound and VCTE examinations were performed by a single trained physician, who was blinded to participants’ clinical and biochemical details, thereby eliminating both assessment bias and interobserver variability. Finally, we excluded patients with important comorbidities (for example, cirrhosis, advanced kidney disease, or cancer), deeming that including patients with such comorbidities might have confounded the interpretation of data.

In conclusion, our cross-sectional study shows that lower plasma adiponectin levels are closely associated with the presence and severity of NAFLD in men with T2DM. Notably, this association remained significant even after adjusting for age, adiposity measures, HOMA-IR score, the *PNPLA3* genetic variant, or other NAFLD-related genetic polymorphisms that confer a greater susceptibility to NAFLD and NASH. Although our findings suggest a possible role of hypoadiponectinemia in the development and progression of NAFLD in patients with T2DM, larger studies are required to further corroborate these results in other patient cohorts and to more thoroughly understand the biological mechanisms underlying this association. We also believe that future studies should better elucidate the specific role of different isoforms of adiponectin in the pathophysiology of NAFLD, as well as the AdipoR1 and AdipoR2 signaling mechanisms involved in the development and progression of NAFLD.

## Supplementary Information

Below is the link to the electronic supplementary material.

Supplementary file1 (DOC 189 KB)
