# Supplementary material for: Association between lower plasma adiponectin levels and higher liver stiffness in type 2 diabetic individuals with nonalcoholic fatty liver disease: an observational cross-sectional study
Source: Hormones (Athens). 2022 Jul 13;21(3):477–86. doi: 10.1007/s42000-022-00387-6 (PMC9464740; doi:10.1007/s42000-022-00387-6)
Supplement: Supplementary file 1 — Supplementary file1 (DOC 189 KB) [file 42000_2022_387_MOESM1_ESM.doc]

**ONLINE-ONLY SUPPLEMENTARY MATERIAL**

**Supplementary Table 1**. Association between high-molecular-weight adiponectin levels and presence and severity of NAFLD (using both ultrasonography and Fibroscan®) in men with type 2 diabetes, after excluding those (n=13) treated with pioglitazone.

|  | **Patients with no hepatic steatosis (n=23)** | **Patients with hepatic steatosis alone (n=26)** | | | **Patients with steatosis and significant fibrosis (n=17)** | | |
| --- | --- | --- | --- | --- | --- | --- | --- |
|  |  | **Odds Ratio(s)** | **95% CI** | ***P* value** | **Odds Ratio(s)** | **95% CI** | ***P* value** |
| **Unadjusted model** |  |  |  |  |  |  |  |
| Decrease in adiponectin (ug/mL) | *Ref.* | 4.54 | 1.67-12.5 | **0.003** | 11.1 | 3.03-33.3 | **<0.001** |
| **Adjusted model 1** |  |  |  |  |  |  |  |
| Decrease in adiponectin (ug/mL) | *Ref.* | 3.04 | 1.01-9.09 | **0.049** | 5.56 | 1.41-20.0 | **0.014** |
| Age (years) | *Ref.* | 0.95 | 0.87-1.04 | 0.241 | 0.94 | 0.85-1.05 | 0.267 |
| Body mass index (kg/m^2^) | *Ref.* | 1.09 | 0.90-1.32 | 0.366 | 1.09 | 0.86-1.38 | 0.468 |
| HOMA-IR score | *Ref.* | 0.97 | 0.56-1.68 | 0.913 | 1.39 | 0.78-2.47 | 0.256 |
| **Adjusted model 2** |  |  |  |  |  |  |  |
| Decrease in adiponectin (ug/mL) | *Ref.* | 3.03 | 1.00-9.10 | **0.051** | 5.55 | 1.39-20.0 | **0.015** |
| Age (years) | *Ref.* | 0.94 | 0.86-1.03 | 0.189 | 0.93 | 0.86-1.04 | 0.220 |
| Body mass index (kg/m^2^) | *Ref.* | 1.10 | 0.90-1.33 | 0.348 | 1.09 | 0.86-1.39 | 0.465 |
| HOMA-IR score | *Ref.* | 0.99 | 0.56-1.75 | 0.967 | 1.42 | 0.78-2.58 | 0.254 |
| *PNPLA3* rs738409 (CC *vs*. CG/GG) | *Ref.* | 1.85 | 0.54-6.38 | 0.328 | 1.79 | 0.42-7.81 | 0.435 |
| **Adjusted model 3** |  |  |  |  |  |  |  |
| Decrease in adiponectin (ug/mL) | *Ref.* | 3.23 | 1.04-10.0 | **0.042** | 6.25 | 1.49-25.0 | **0.012** |
| Age (years) | *Ref.* | 0.95 | 0.87-1.03 | 0.226 | 0.94 | 0.86-1.04 | 0.253 |
| Body mass index (kg/m^2^) | *Ref.* | 1.12 | 0.91-1.37 | 0.275 | 1.11 | 0.86-1.41 | 0.432 |
| HOMA-IR score | *Ref.* | 0.92 | 0.53-1.60 | 0.774 | 1.33 | 0.75-2.36 | 0.326 |
| *TM6SF2* rs58542926 (CC *vs*. CT) | *Ref.* | 2.78 | 0.35-21.9 | 0.332 | 1.29 | 0.07-23.9 | 0.865 |
| **Adjusted model 4** |  |  |  |  |  |  |  |
| Decrease in adiponectin (ug/mL) | *Ref.* | 3.22 | 1.03-10.0 | **0.044** | 5.56 | 1.41-25.0 | **0.015** |
| Age (years) | *Ref.* | 0.95 | 0.87-1.04 | 0.269 | 0.94 | 0.85-1.05 | 0.279 |
| Body mass index (kg/m^2^) | *Ref.* | 1.10 | 0.90-1.33 | 0.346 | 1.09 | 0.86-1.38 | 0.475 |
| HOMA-IR score | *Ref.* | 1.97 | 0.56-1.68 | 0.912 | 1.39 | 0.79-2.49 | 0.254 |
| *MBOAT7* rs641738 (CC *vs*. CT/TT) | *Ref.* | 1.31 | 0.47-3.66 | 0.609 | 1.07 | 0.28-4.19 | 0.917 |

Sample size, *n*=66 unless where indicated. Data are expressed as odds ratio and 95% confidence intervals (CI) as tested by multinomial logistic regression analysis. The dependent variable for all multinomial logistic regression models was the presence and severity of NAFLD (using both ultrasonography and Fibroscan), categorized as follows: patients without hepatic steatosis on ultrasound (the reference group), patients with hepatic steatosis alone and patients with steatosis and coexisting significant fibrosis. Plasma Adiponectin and HOMA-IR were logarithmically transformed before analysis.

*Abbreviations*: HOMA-IR: homeostasis model assessment-insulin resistance; MBOAT7, membrane bound O-acyltransferase domain containing 7; PNPLA3, patatin-like phospholipase domain-containing protein 3; Ref*.*, reference category; TM6SF2, transmembrane 6 superfamily member 2.

**Supplementary Table 2**. Association between high-molecular-weight adiponectin levels and presence and severity of NAFLD (using both ultrasonography and Fibroscan®) in men with type 2 diabetes, after excluding those (n=12) treated with SGLT-2 inhibitors.

|  | **Patients with no hepatic steatosis (n=27)** | **Patients with hepatic steatosis alone (n=25)** | | | **Patients with steatosis and significant fibrosis (n=15)** | | |
| --- | --- | --- | --- | --- | --- | --- | --- |
|  |  | **Odds Ratio(s)** | **95% CI** | ***P* value** | **Odds Ratio(s)** | **95% CI** | ***P* value** |
| **Unadjusted model** |  |  |  |  |  |  |  |
| Decrease in adiponectin (ug/mL) | *Ref.* | 3.23 | 1.39-7.14 | **0.006** | 6.25 | 2.12-17.9 | **0.001** |
| **Adjusted model 1** |  |  |  |  |  |  |  |
| Decrease in adiponectin (ug/mL) | *Ref.* | 2.56 | 1.03-6.25 | **0.044** | 4.00 | 1.11-14.9 | **0.035** |
| Age (years) | *Ref.* | 0.96 | 0.88-1.04 | 0.291 | 0.98 | 0.88-1.08 | 0.685 |
| Body mass index (kg/m^2^) | *Ref.* | 1.18 | 0.95-1.48 | 0.140 | 1.20 | 0.91-1.58 | 0.192 |
| HOMA-IR score | *Ref.* | 0.91 | 0.50-1.64 | 0.744 | 1.48 | 0.80-2.72 | 0.209 |
| **Adjusted model 2** |  |  |  |  |  |  |  |
| Decrease in adiponectin (ug/mL) | *Ref.* | 2.56 | 1.02-6.41 | **0.043** | 4.76 | 1.18-20.0 | **0.015** |
| Age (years) | *Ref.* | 0.96 | 0.89-1.04 | 0.342 | 0.98 | 0.89-1.09 | 0.776 |
| Body mass index (kg/m^2^) | *Ref.* | 1.18 | 0.94-1.49 | 0.148 | 1.24 | 0.92-1.66 | 0.156 |
| HOMA-IR score | *Ref.* | 0.91 | 0.49-1.69 | 0.763 | 1.44 | 0.75-2.75 | 0.276 |
| *PNPLA3* rs738409 (CC *vs*. CG/GG) | *Ref.* | 1.44 | 0.49-4.21 | 0.500 | 2.17 | 0.51-9.20 | 0.292 |
| **Adjusted model 3** |  |  |  |  |  |  |  |
| Decrease in adiponectin (ug/mL) | *Ref.* | 2.70 | 1.05-6.76 | **0.039** | 4.42 | 1.17-16.7 | **0.029** |
| Age (years) | *Ref.* | 0.95 | 0.87-1.03 | 0.239 | 0.98 | 0.88-1.08 | 0.656 |
| Body mass index (kg/m^2^) | *Ref.* | 1.20 | 0.96-1.50 | 0.113 | 1.22 | 0.93-1.60 | 0.165 |
| HOMA-IR score | *Ref.* | 0.86 | 0.48-1.55 | 0.624 | 1.41 | 0.78-2.56 | 0.257 |
| *TM6SF2* rs58542926 (CC *vs*. CT) | *Ref.* | 3.23 | 0.39-26.6 | 0.275 | 1.52 | 0.09-26.9 | 0.775 |
| **Adjusted model 4** |  |  |  |  |  |  |  |
| Decrease in adiponectin (ug/mL) | *Ref.* | 2.56 | 1.02-6.29 | **0.044** | 4.11 | 1.09-15.4 | **0.036** |
| Age (years) | *Ref.* | 0.96 | 0.88-1.04 | 0.292 | 0.98 | 0.88-1.08 | 0.688 |
| Body mass index (kg/m^2^) | *Ref.* | 1.18 | 0.95-1.48 | 0.141 | 1.20 | 0.91-1.59 | 0.195 |
| HOMA-IR score | *Ref.* | 0.91 | 0.50-1.64 | 0.744 | 1.48 | 0.80-2.72 | 0.210 |
| *MBOAT7* rs641738 (CC *vs*. CT/TT) | *Ref.* | 0.99 | 0.37-2.67 | 0.987 | 1.02 | 0.27-3.95 | 0.970 |

Sample size, *n*=67 unless where indicated. Data are expressed as odds ratio and 95% confidence intervals (CI) as tested by multinomial logistic regression analysis. The dependent variable for all multinomial logistic regression models was the presence and severity of NAFLD (using both ultrasonography and Fibroscan), categorized as follows: patients without hepatic steatosis on ultrasound (the reference group), patients with hepatic steatosis alone and patients with steatosis and coexisting significant fibrosis. Plasma Adiponectin and HOMA-IR were logarithmically transformed before analysis.

*Abbreviations*: HOMA-IR: homeostasis model assessment-insulin resistance; MBOAT7, membrane bound O-acyltransferase domain containing 7; PNPLA3, patatin-like phospholipase domain-containing protein 3; Ref*.*, reference category; TM6SF2, transmembrane 6 superfamily member 2.

**Supplementary Table 3**. Association between high-molecular-weight adiponectin levels and presence and severity of NAFLD (using both ultrasonography and Fibroscan®) in men with type 2 diabetes, after excluding those (n=21) treated with GLP-1 receptor agonists.

|  | **Patients with no hepatic steatosis (n=22)** | **Patients with hepatic steatosis alone (n=23)** | | | **Patients with steatosis and significant fibrosis (n=13)** | | |
| --- | --- | --- | --- | --- | --- | --- | --- |
|  |  | **Odds Ratio(s)** | **95% CI** | ***P* value** | **Odds Ratio(s)** | **95% CI** | ***P* value** |
| **Unadjusted model** |  |  |  |  |  |  |  |
| Decrease in adiponectin (ug/mL) | *Ref.* | 2.27 | 0.96-5.55 | 0.063 | 5.26 | 1.69-15.9 | **0.004** |
| **Adjusted model 1** |  |  |  |  |  |  |  |
| Decrease in adiponectin (ug/mL) | *Ref.* | 1.75 | 0.66-4.76 | 0.259 | 3.31 | 1.02-12.5 | **0.047** |
| Age (years) | *Ref.* | 0.93 | 0.86-1.01 | 0.091 | 0.95 | 0.86-1.05 | 0.281 |
| Body mass index (kg/m^2^) | *Ref.* | 1.11 | 0.91-1.36 | 0.310 | 1.11 | 0.87-1.42 | 0.411 |
| HOMA-IR score | *Ref.* | 0.99 | 0.54-1.81 | 0.969 | 1.26 | 0.65-2.44 | 0.491 |
| **Adjusted model 2** |  |  |  |  |  |  |  |
| Decrease in adiponectin (ug/mL) | *Ref.* | 1.64 | 0.61-4.54 | 0.325 | 3.22 | 1.01-11.0 | **0.049** |
| Age (years) | *Ref.* | 0.92 | 0.85-1.01 | 0.065 | 0.94 | 0.85-1.04 | 0.244 |
| Body mass index (kg/m^2^) | *Ref.* | 1.13 | 0.92-1.39 | 0.243 | 1.12 | 0.87-1.43 | 0.382 |
| HOMA-IR score | *Ref.* | 1.05 | 0.56-1.96 | 0.881 | 1.31 | 0.67-2.57 | 0.430 |
| *PNPLA3* rs738409 (CC *vs*. CG/GG) | *Ref.* | 2.26 | 0.63-8.09 | 0.209 | 1.55 | 0.33-7.28 | 0.578 |
| **Adjusted model 3** |  |  |  |  |  |  |  |
| Decrease in adiponectin (ug/mL) | *Ref.* | 1.89 | 0.69-5.26 | 0.214 | 3.70 | 1.02-12.2 | **0.047** |
| Age (years) | *Ref.* | 0.93 | 0.85-1.01 | 0.083 | 0.95 | 0.86-1.04 | 0.266 |
| Body mass index (kg/m^2^) | *Ref.* | 1.14 | 0.93-1.42 | 0.216 | 1.13 | 0.87-1.46 | 0.355 |
| HOMA-IR score | *Ref.* | 0.91 | 0.49-1.69 | 0.768 | 1.17 | 0.60-2.29 | 0.639 |
| *TM6SF2* rs58542926 (CC *vs*. CT) | *Ref.* | 4.19 | 0.46-38.4 | 0.205 | 1.73 | 0.09-32.9 | 0.714 |
| **Adjusted model 4** |  |  |  |  |  |  |  |
| Decrease in adiponectin (ug/mL) | *Ref.* | 1.78 | 0.65-4.76 | 0.257 | 3.03 | 0.88-10.0 | 0.078 |
| Age (years) | *Ref.* | 0.93 | 0.85-1.01 | 0.088 | 0.94 | 0.85-1.04 | 0.226 |
| Body mass index (kg/m^2^) | *Ref.* | 1.12 | 0.91-1.38 | 0.251 | 1.09 | 0.85-1.40 | 0.484 |
| HOMA-IR score | *Ref.* | 0.93 | 0.49-1.75 | 0.832 | 1.32 | 0.66-2.63 | 0.430 |
| *MBOAT7* rs641738 (CC *vs*. CT/TT) | *Ref.* | 1.66 | 0.59-4.66 | 0.338 | 0.76 | 0.20-2.91 | 0.688 |

Sample size, *n*=58 unless where indicated. Data are expressed as odds ratio and 95% confidence intervals (CI) as tested by multinomial logistic regression analysis. The dependent variable for all multinomial logistic regression models was the presence and severity of NAFLD (using both ultrasonography and Fibroscan), categorized as follows: patients without hepatic steatosis on ultrasound (the reference group), patients with hepatic steatosis alone and patients with steatosis and coexisting significant fibrosis. Plasma Adiponectin and HOMA-IR were logarithmically transformed before analysis.

*Abbreviations*: HOMA-IR: homeostasis model assessment-insulin resistance; MBOAT7, membrane bound O-acyltransferase domain containing 7; PNPLA3, patatin-like phospholipase domain-containing protein 3; Ref*.*, reference category; TM6SF2, transmembrane 6 superfamily member 2.

**Supplementary Table 4**. Adjusted associations between high-molecular-weight adiponectin levels and presence and severity of NAFLD in patients with type 2 diabetes after adjustment for other single genotyped NAFLD-related polymorphisms.

|  | **Patients without hepatic steatosis (*n*=28)** | **Patients with**  **hepatic steatosis alone (*n*=32)** | | | **Patients with**  **steatosis and significant fibrosis (*n*=19)** | | |
| --- | --- | --- | --- | --- | --- | --- | --- |
|  |  | **Odds Ratio(s)** | **95% CI** | ***P*-value** | **Odds Ratio(s)** | **95% CI** | ***P*-value** |
| **Adjusted model 1** |  |  |  |  |  |  |  |
| Decrease in adiponectin (ug/mL) | *Ref.* | 2.22 | 0.92- 5.56 | 0.079 | 3.84 | 1.23-12.5 | **0.021** |
| Age (years) | *Ref.* | 0.94 | 0.88-1.02 | 0.130 | 0.94 | 0.86-1.03 | 0.176 |
| Body mass index (kg/m^2^) | *Ref.* | 1.14 | 0.95-1.37 | 0.171 | 1.13 | 0.90-1.42 | 0.293 |
| HOMA-IR score | *Ref.* | 0.98 | 0.57-1.71 | 0.955 | 1.48 | 0.83-2.65 | 0.181 |
| *GCKR* rs1260326 (CC *vs*. CT/TT) | *Ref.* | 0.73 | 0.30-1.76 | 0.479 | 1.14 | 0.33-3.92 | 0.837 |
| **Adjusted model 2** |  |  |  |  |  |  |  |
| Decrease in adiponectin (ug/mL) | *Ref.* | 2.38 | 1.02-5.88 | **0.045** | 3.70 | 1.20-11.2 | **0.022** |
| Age (years) | *Ref.* | 0.95 | 0.88-1.03 | 0.143 | 0.94 | 0.86-1.03 | 0.197 |
| Body mass index (kg/m^2^) | *Ref.* | 1.13 | 0.94-1.37 | 0.200 | 1.13 | 0.89-1.43 | 0.290 |
| HOMA-IR score | *Ref.* | 1.02 | 0.59-1.76 | 0.934 | 1.48 | 0.84-2.63 | 0.176 |
| *SOD2* rs4880 (AA *vs*. AG/GG) | *Ref.* | 0.94 | 0.38-2.33 | 0.896 | 0.86 | 0.29-2.54 | 0.790 |
| **Adjusted model 3** |  |  |  |  |  |  |  |
| Decrease in adiponectin (ug/mL) | *Ref.* | 2.63 | 1.10-6.25 | **0.029** | 3.23 | 1.05-10.0 | **0.041** |
| Age (years) | *Ref.* | 0.95 | 0.88-1.02 | 0.158 | 0.94 | 0.86-1.03 | 0.206 |
| Body mass index (kg/m^2^) | *Ref.* | 1.12 | 0.92-1.35 | 0.258 | 1.14 | 0.90-1.44 | 0.292 |
| HOMA-IR score | *Ref.* | 1.04 | 0.60-1.80 | 0.891 | 1.51 | 0.85-2.68 | 0.161 |
| *ELOVL2* rs2236212 (CC vs. CG/GG) | *Ref.* | 0.57 | 0.24-1.35 | 0.201 | 1.31 | 0.42-4.06 | 0.644 |
| **Adjusted model 4** |  |  |  |  |  |  |  |
| Decrease in adiponectin (ug/mL) | *Ref.* | 2.44 | 1.03-5.55 | **0.043** | 3.57 | 1.20-11.1 | **0.022** |
| Age (years) | *Ref.* | 0.95 | 0.88-1.02 | 0.142 | 0.94 | 0.86-1.03 | 0.195 |
| Body mass index (kg/m^2^) | *Ref.* | 1.13 | 0.94-1.36 | 0.186 | 1.14 | 0.91-1.43 | 0.262 |
| HOMA-IR score | *Ref.* | 1.03 | 0.59-1.77 | 0.927 | 1.45 | 0.82-2.57 | 0.199 |
| *LPIN1* rs13412852 (CC *vs*. CT/TT) | *Ref.* | 0.96 | 0.42-2.23 | 0.932 | 1.30 | 0.47-3.62 | 0.614 |
| **Adjusted model 5** |  |  |  |  |  |  |  |
| Decrease in adiponectin (ug/mL) | *Ref.* | 2.32 | 1.00-5.56 | 0.050 | 3.58 | 1.18-11.0 | **0.025** |
| Age (years) | *Ref.* | 0.94 | 0.86-1.01 | 0.097 | 0.93 | 0.85-1.03 | 0.160 |
| Body mass index (kg/m^2^) | *Ref.* | 1.12 | 0.93-1.36 | 0.242 | 1.12 | 0.89-1.42 | 0.330 |
| HOMA-IR score | *Ref.* | 1.11 | 0.61-1.99 | 0.735 | 1.54 | 0.83-2.87 | 0.170 |
| *MTTP* rs1800591 (GG *vs*. GT/TT) | *Ref.* | 0.46 | 0.17-1.22 | 0.119 | 0.73 | 0.23-2.25 | 0.579 |
| **Adjusted model 6** |  |  |  |  |  |  |  |
| Decrease in adiponectin (ug/mL) | *Ref.* | 2.38 | 1.03-5.55 | **0.043** | 3.57 | 1.22-11.2 | **0.021** |
| Age (years) | *Ref.* | 0.95 | 0.88-1.02 | 0.166 | 0.94 | 0.86-1.03 | 0.208 |
| Body mass index (kg/m^2^) | *Ref.* | 1.14 | 0.94-1.37 | 0.182 | 1.14 | 0.91-1.44 | 0.250 |
| HOMA-IR score | *Ref.* | 1.04 | 0.61-1.78 | 0.885 | 1.48 | 0.84-2.59 | 0.175 |
| *FADS2* rs1535 (AA *vs*. AG/GG) | *Ref.* | 0.69 | 0.26-1.85 | 0.463 | 1.00 | 0.31-3.29 | 0.997 |

Sample size, *n*=79 unless where indicated. Data are expressed as odds ratio and 95% confidence intervals (CI) as tested by multinomial logistic regression analysis. The dependent variable for all multinomial logistic regression models was the presence and severity of NAFLD, categorized as follows: patients without hepatic steatosis on ultrasound (the reference group), patients with hepatic steatosis alone and patients with steatosis and coexisting significant fibrosis on VCTE. Plasma adiponectin and HOMA-IR values were logarithmically transformed before analysis. The impact of each genetic variant on the presence and severity of NAFLD was assessed using dominant genetic models.

*Abbreviations*: GCKR, glucokinase regulatory protein; EVOLV2, elongation of very-long-chain fatty acids-like 2; FADS2, fatty acid desaturase 2; HOMA-IR: homeostasis model assessment-insulin resistance; LPIN1, lipin 1; MTTP, microsomal triglyceride transfer protein; SOD2, superoxide dismutase 2.
